# More than fishing in the dark: PCR of a dispersed sequence produces simple but ultrasensitive *Wolbachia* detection

**DOI:** 10.1186/1471-2180-14-121

**Published:** 2014-05-12

**Authors:** Daniela I Schneider, Lisa Klasson, Anders E Lind, Wolfgang J Miller

**Affiliations:** 1 Laboratory of Genome Dynamics, Department of Cell and Developmental Biology, Center for Anatomy and Cell Biology, Medical University of Vienna, Waehringerstrasse 10, Vienna 1090, Austria; 2 Department of Molecular Evolution, Cell and Molecular Biology, Science for Life Laboratory, Biomedical Centre, Uppsala, Sweden

**Keywords:** *Wolbachia*, *Drosophila*, *Glossina*, Hybrid, High- and low-titer endosymbiont infection, Limit of detection, *A*-supergroup *r*epeat *m*otif (ARM)

## Abstract

**Background:**

Detecting intracellular bacterial symbionts can be challenging when they persist at very low densities. *Wolbachia*, a widespread bacterial endosymbiont of invertebrates, is particularly challenging. Although it persists at high titers in many species, in others its densities are far below the detection limit of classic end-point Polymerase Chain Reaction (PCR). These low-titer infections can be reliably detected by combining PCR with DNA hybridization, but less elaborate strategies based on end-point PCR alone have proven less sensitive or less general.

**Results:**

We introduce a multicopy PCR target that allows fast and reliable detection of A-supergroup *Wolbachia* - even at low infection titers - with standard end-point PCR. The target is a multicopy motif (designated ARM: *A*-supergroup *r*epeat *m*otif) discovered in the genome of *w*Mel (the *Wolbachia* in *Drosophila melanogaster*). ARM is found in at least seven other *Wolbachia* A-supergroup strains infecting various *Drosophila*, the wasp *Muscidifurax* and the tsetse fly *Glossina*. We demonstrate that end-point PCR targeting ARM can reliably detect both high- and low-titer *Wolbachia* infections in *Drosophila*, *Glossina* and interspecific hybrids.

**Conclusions:**

Simple end-point PCR of ARM facilitates detection of low-titer *Wolbachia* A-supergroup infections. Detecting these infections previously required more elaborate procedures. Our ARM target seems to be a general feature of *Wolbachia* A-supergroup genomes, unlike other multicopy markers such as insertion sequences (IS).

## Background

Detecting endosymbionts such as the widespread alphaproteobacterium *Wolbachia* in its host cell environment requires reliable and ideally simple but still sensitive molecular marker systems. When such bacteria are present at high titers, classic end-point PCR is sufficient to unambiguously determine infection status of an unknown specimen. Particularly for *Wolbachia*, a quite comprehensive set of diagnostic PCR markers has been developed and applied successfully. The most commonly used among these makers is the multi locus sequence typing (MLST) system [1-3] and the four hypervariable regions (HVRs) of the *Wolbachia* outer surface protein gene *wsp*[4,5]. Both MLST, comprising a set of five singlecopy *Wolbachia* genes, and the *wsp* locus were demonstrated to be highly useful for *Wolbachia* infection determination and consequent diversity assessment. However, those marker systems are limited if the endosymbiont persists at very low titers within the host, either only during a certain ontogenetic stage [6] or throughout all life stages. In both cases proper detection of the endosymbiont is hindered and this points towards the need of an alternative strategy for efficient, robust and fast *Wolbachia* detection. One approach to address this issue is to use multicopy *Wolbachia* gene markers for PCR analyses. Particularly insertion sequences (IS; [7,8]) represent a good strategy to increase the detection threshold [9,10]. However, this approach relies on the conservation of such elements and their copy-numbers in diverse strains, which might not be the case over longer evolutionary distances due to the mobile nature of these elements. Another approach to cope with the detection problem introduced by low-titer infections is ‘nested PCR’. This method might help to increase the detection threshold but is also highly prone to contamination [6]. A third strategy combines standard PCR with consequent hybridization [6,11,12], which increases overall detection limit by four orders of magnitude [6]. On the other hand, this is an elaborate and time-consuming technique. Hence, we set out to find a more sensitive marker for detection of low-titer *Wolbachia* infections using standard PCR and identified ARM as such a simple but ‘ultra-sensitive’ marker for A-supergroup *Wolbachia*.

## Results and discussion

### Identification of a multicopy marker associated with tandem repeats in A-supergroup Wolbachia genomes (ARM)

**Figure 1:**
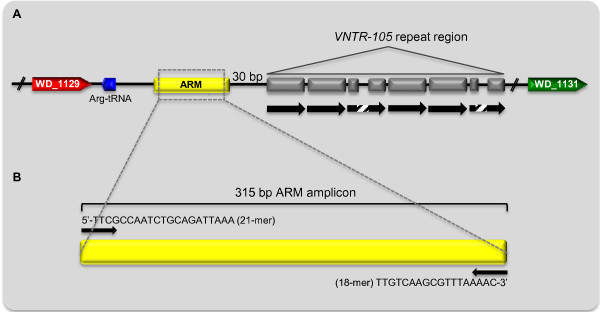
**Schematic presentation of ARM. (A)** Position of ARM in association with *VNTR-105* locus plus flanking regions in the *w*Mel genome (GenBank NC_002978). Scheme for *VNTR-105* repeat region was adapted from [13] (see this publication for detailed description of *VNTR-105* structural features). Black arrows indicate the full 105 bp core repeat segment. Dashed box represents a disrupted segment. ARM (highlighted in yellow) is located within the intergenic region containing the *VNTR-105* repeat region. ARM plus repeat region are flanked by WD_1129 (red; NADH-ubiquinone oxidoreductase, putative) on the 5’-prime end and WD_1131 (green; conserved hypothetical protein, degenerate) on the 3’-prime end. **(B)** Detailed scheme of ARM. The 315 bp PCR amplicon is generated by primer ARM-F (21-mer) and ARM-R (18-mer). Both primers are displayed above and below the PCR amplicon (indicated in yellow).

To find a marker that serves a highly sensitive detection method of low-titer *Wolbachia* strains we identified multicopy regions in the A-supergroup *w*Mel genome (*Wolbachi*a of *Drosophila melanogaster*; GenBank NC_002978). An intergenic region of 440 bp associated with the recently described hypervariable tandem repeat region (Figure 1; [13]) was the most promising candidate, hereafter called ARM (*A*-supergroup *r*epeat *m*otif) as it was found in 24 almost identical copies dispersed throughout the *w*Mel genome (Additional file 1). However, for a marker to be useful as a general tool it also needs to be conserved and present in multiple copies in other strains and we therefore used the *w*Mel repeat sequence to search an additional 13 draft and complete *Wolbachia* genomes from four different *Wolbachia* supergroups for the same sequence. We were able to identify the presence of the repeat in seven A-supergroup *Wolbachia* genomes (*w*Ha, *w*Ri, *w*Wil, *w*Ana, *w*Uni, *w*Suzi and *w*Gmm; see Table 1), albeit in variable copy numbers. In the *Drosophila* associated *Wolbachia* strains, the copy numbers were around 20 per genome (Table 1), whereas the other two A-supergroup genomes (*w*Uni and *w*Gmm) contained about half the amount of copies. Low number of hits in *w*Uni is most likely explained by the incomplete status of the genome resulting in an underestimation of the actual copy number. In the B- (*w*No, *w*VitB, *w*Pip), C- (*w*Oo, *w*Ov), and D-supergroup (*w*Bm) genomes, ARM was not found. Even though some of the genomes in supergroups B, C, and D are incomplete, the total absence of the repeat in all genomes from these supergroups suggests that this motif might be *Wolbachia* A-supergroup specific. Additionally, VNTR-tandem repeats associated with ARM in A-supergroup infections are also absent from genomes of B- to D-supergroups, further indicating that this feature might indeed be A-supergroup specific.

**Table 1:** **Number of matches to ARM in complete and draft *Wolbachia *** **genomes**

***Wolbachia***	**Supergroup**	**Host**	**Number of matches to *ARM***	**GenBank references**
***w*** **Mel**	A	*Drosophila melanogaster*	24	NC_002978; [8]
***w*** **Ha**	A	*Drosophila simulans*	23	CP003884; [23]
***w*** **Ri**	A	*Drosophila simulans*	21	NC_012416; [22]
***w*** **Wil**	A	*Drosophila willistoni*	17^a^	ASM15358v1; TSC#14030-0811.24
***w*** **Ana**	A	*Drosophila ananassae*	20^a^	ASM16747v1; [24]
***w*** **Uni**	A	*Muscidifax uniraptor*	7^a^	wUni_1.0; [22]
***w*** **Suzi**	A	*Drosophila suzukii*	23^a^	CAOU02000000; [25]
***w*** **Gmm**	A	*Glossina morsitans morsitans*	20^a^	[14]
***w*** **No**	B	*Drosophila simulans*	0^b^	CP003883; [23]
***w*** **VitB**	B	*Nasonia vitripennis*	0^b^	WVB_1.0; [26]
***w*** **Pip**	B	*Culex quinquefasciatus*	0^b^	NC_010981.1; [27]
***w*** **Oo**	C	*Onchocerca ochengi*	0^b^	NC_018267.1; [28]
***w*** **Ov**	C	*Onchocerca volvulus*	0^b^	ASM33837v1; [29]
***w*** **Bm**	D	*Brugia malayi*	0^b^	NC_006833.1; [30]

Number of matches in column four refer to hits of the 315 bp ARM-PCR amplicon in the searched *Wolbachia* genomes. Hits were produced using the blastn algorithm (megablast) with match/mismatch scores 1,-2. *Wolbachia* strains are organized by supergroup (column two). Matches to ARM were only found within the A-supergroup. ^a^Minimum number of ARMs in the corresponding genome. Exact number cannot be given due to the lack of a complete genome. ^b^Refers to no similarity detected between ARM and searched genome (complete/draft).

### ARM facilitates detection of low-titer Wolbachia from A-supergoup

**Figure 2:**
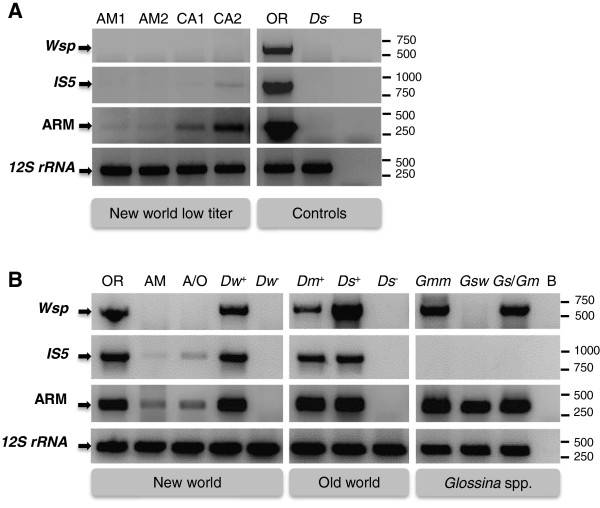
**Comparison of *Wolbachia *marker sensitivity by PCR. (A)** The three *Wolbachia* markers *wsp*, *IS5* and ARM were tested on the following specimens: New world *Drosophila* species from the *Drosophila willistoni* group including *D. paulistorum* Amazonian (AM1, AM2), and Centroamerican (CA1, CA2) semispecies. Orinocan semispecies (OR) served as *Wolbachia* positive control; *Ds*^-^ as *Wolbachia* negative control. B = blank. Quality of DNA was assessed with universal primer set 12SCFR, 12SCRR targeting the mitochondrial *12S rRNA* gene [20,21]. Expected amplicon sizes for *Wolbachia* positive control (OR) are 631 bp (*wsp*), 752 bp (*IS5*), 315 bp (ARM) and 399 bp (*12S rRNA*). **(B)** Same markers as above were tested on additional samples including hybrids: A/O hybrid plus parents AM and OR; *Glossina Gs*/*Gm* hybrid plus parental strains *Gsw* and *Gmm* (Additional file 2). *Drosophila* New world members include *D. willistoni Dw*^+^ and *Dw*^-^. Old world species are *D. melanogaster Dm*^+^; *D. simulans Ds*^+^ and *Ds*^-^. B = blank. Note: *IS5* primer set does not produce amplicons in all three *Glossina* samples due to complete absence of this IS element in symbionts of tsetse flies (see discussion).

ARM-targeting primer were tested via end-point PCR screen on DNA from high- and low-titer *Wolbachia* infections in *Drosophila* and *Glossina* (tsetse fly) species (Additional file 2). As shown in Figure 2, the classic *Wolbachia* singlecopy gene marker *wsp* (Wolbachia outer surface protein gene) is only applicable for samples with high-titer infections, since *Wolbachia* was only detected in high-titer *D. paulistorum* Orinocan semispecies (OR, Figure 2A) as well as in *D. willistoni* (*Dw*^+^, Figure 2B), *D. melanogaster* (*Dm*^+^, Figure 2B), *D. simulans* (*Ds*^+^, Figure 2B) and *Glossina morsitans morsitans* (*Gmm*, Figure 2B). The *wsp* primer failed to detect *Wolbachia* in low-titer strains like *D. paulistorum* Amazonian (AM) and Centroamerican (CA) semispecies plus *Glossina swynnertoni* (Figure 2A,B), indicating that a singlecopy gene like *wsp* is not suited for tracking low-titer infections. As multicopy gene markers like insertion sequences (IS) can be used to increase the detection limit, we ran PCR using primer for Insertion Sequence 5 (*IS5*; [8-10] on the same sample set. We observed increased sensitivity compared to *wsp*-PCR since *Wolbachia* was detected in low-titer CA2 (Figure 2A) and in the A/O hybrid samples. However, *IS5* primer failed at amplifying the target sequence in all three *Glossina* samples (*Gmm*, *Gsw* and *Gs*/*Gm* hybrid; Figure 2B) despite the overall high *Wolbachia* titer in *Gmm*[12].

We have recently shown that *Wolbachia* titers increase in *D. paulistorum*[11] and *Glossina*[12] hybrid backgrounds, which should significantly facilitate detection and strain characterization. Such titer increase was sufficient to detect *Wolbachia* with the *IS5* primer set in A/O hybrids, but the low-titer *Wolbachia* infection in the AM mother still remained undetected (Figure 2B). Failure of *IS5*-amplification in the *Gs*/*Gm* hybrid plus parents is explained by lacking homology between primer sequences and target, as no matches with the *IS5* primer sequence were found in the *w*Gmm genome [14]. This finding implies that *IS5* is not suitable as a general *Wolbachia* A-supergroup marker.

Figure 2A and B show that the ARM-marker system can be applied to address aforementioned problems arising with *wsp* and *IS5* primer: sensitivity during PCR is increased significantly and all tested A-supergroup infections are unambiguously detected. *Wolbachia* was traced in all low-titer New world *Drosophila* species (AM1, AM2; CA1, CA2) plus the A/O hybrid. In contrast to *IS5*, the ARM primer set amplified *Wolbachia* from all three *Glossina* samples (*Gmm*, *Gsw* and *Gs*/*Gm* hybrid). As anticipated, all samples from high-titer *Wolbachia* infections (OR, *Dw*^*+*^, *Dm*^+^, *Ds*^+^) showed bright bands with ARM, whereas *Wolbachia*-uninfected specimens (*Dw*^-^, *Ds*^-^) did not (Figure 2A,B). This argues for a high specificity of the ARM primer and against mis-amplification of a random host target rather than the specific symbiont target site.

## Conclusions

We suggest that the new multicopy *Wolbachia* A-supergroup marker can be used as an ‘ultra-sensitive’ tool to trace low-titer infections by means of classic end-point PCR. First, ARM has the advantage of higher sensitivity compared to classic singlecopy *Wolbachia* markers like *wsp* and thus improves detection limit significantly. Particularly, ARM-PCR can be easily applied to screen larger numbers of untyped DNA specimens, even of low quality arising from long-term storage and/or storage in inappropriate media, from laboratory stocks or samples directly from nature. This is of pivotal interest since classical detection tools might yield false negatives when examining species harboring *Wolbachia* at very low densities, and thereby lead to underestimating natural prevalence of A-supergroup infections. Given that 80% of the Dipteran infections are supergroup A [15], our new method will significantly facilitate and improve the sensitivity of such surveys. In addition our approach is an advantage over the classic *IS5*-marker, which fails in *Wolbachia* from the tsetse fly *Glossina*. Taken together, we show that a *Wolbachia* sequence motif found in multiple copies associated with the *VNTR* loci facilitates reliable *Wolbachia* screening of samples from low-titer infections and might thus serve as a great tool for the *Wolbachia* research community. Furthermore a similar approach might be applied to detect other symbionts such as *Sodalis glossinidius* (secondary symbiont of *Glossina*) and the primary symbiont *Candidatus* Sodalis pierantonius str. SOPE of the weevil *Sitophilus orizae*. Both symbiont genomes exhibit more than 20% of repetitive DNA rendering them appropriate candidates for repeat-based PCR analysis [16,17]. However, we anticipate that such a method reaches its limit when dealing with symbiont genomes, which have become highly streamlined in the course of tight host-symbiont coevolution.

## Methods

### Drosophila and Glossina strains plus hybrid samples

*Drosophila* specimens included members of New world and Old world clades (Additional file 2). Representatives of the new world clade were *Drosophila paulistorum* semispecies AM, CA and OR, together with *Wolbachia-*infected (*Dw*^*+*^) and -uninfected (*Dw*^*-*^) *D. willistoni* (see Additional file 2 for details). The Old world clade was represented by *Wolbachia-*infected *D. melanogaster* (*Dm*^+^) and *Wolbachia-*infected (*Ds*^+^) and uninfected (*Ds*^-^) *D. simulans* (Additional file 2). Additionally, the tsetse fly species *Glossina swynnertoni* and *G. morsitans morsitans* (genus *Glossina*, superfamily Hippoboscoidea) and hybrids from *D. paulistorum* (A/O) and *Glossina* (*Gs*/*Gm*) were included (Additional file 2). Detailed descriptions of establishing hybrid samples can be found in [11,12]. *Drosophila* strains are permanently maintained in the Laboratory of Genome Dynamics in Vienna, *Glossina* colonies are kept at the Insect Pest Control Laboratory, Joint FAO/IAEA Division of Nuclear Techniques in Food and Agriculture, Vienna, Austria.

### Analysis of complete and draft Wolbachia genomes for candidate marker loci and primer design

Candidate multicopy marker regions were identified by running nucmer and repeat-match from the MUMmer 3 package [18] on the *w*Mel genome (*Wolbachia*, endosymbiont of *Drosophila melanogaster*; GenBank reference NC_002978). Searches were performed with the megablast algorithm using default settings against 14 *Wolbachia* genomes present in GenBank (see Table 1; http://www.ncbi.nlm.nih.gov) and other analyses were performed using Geneious 5.6.6 software (Biomatters, New Zealand).

### Diagnostic wsp-, IS5-, ARM- and 12S rRNA-PCR

Primer pairs for diagnostic *wsp*-PCR were taken from [19] and the corresponding PCR set-up is described in [11]. Primers and PCR profile for *IS5* can be found in [9]. We designed the following primer set targeting ARM: ARM-F 5’-TTCGCCAATCTGCAGATTAAA-3’ and ARM-R 5’-GTTTTAAACGCTTGACAA-3’. Both primers are positioned in the flanking regions of the *VNTR-105* locus in *w*Mel [9,13], and produce an amplicon of 315 bp constant size. Composition of the locus is shown in Figure 1. Diagnostic ARM-PCR was performed in 20 μl reactions containing 1x reaction buffer, 3.0 mM MgCl_2_, 0.4 μM of forward and reverse primer, 35 μM dNTPs, 0.4 U of *Taq* Polymerase (Promega) and 2 μl of DNA template. PCR was performed using a profile of 2 min initial denaturation at 94°C followed by 30 cycles consisting of 45 sec denaturation at 94°C, 45 sec annealing at 55°C, and 1 min extension at 72°C. Final extension was performed for 10 min at 72°C. In order to assess DNA quality, we amplified part of the mitochondrial *12S rRNA* gene with primer set 12SCFR 5′-GAGAGTGACGGGCGATATGT-3′ and 12SCRR 5′-AAACCAGGATTAGATACCCTATTAT-3′ [20]. PCR conditions are outlined in [21]. PCR amplicons were examined using gel-electrophoresis on a 1% agarose gel pre-stained with 0.05 mg ethidium bromide.

## Ethics statement

This study did not involve any subjects and materials that require approval by an ethics committee (human, vertebrate, regulated invertebrates). No genetically modified organisms were part of this study.

## Abbreviations

VNTR: Variable number of tandem repeats; wsp: *Wolbachia* outer surface protein gene; IS5: Insertion sequence element 5; ARM: A-supergroup repeat motif.

## Competing interests

The authors declare that they have no competing interests.

## Authors’ contributions

DIS and WJM conceived the study. DIS, LK, AEL and WJM designed and performed the experiments. WJM provided material. DIS, LK, AEL and WJM analyzed the data. DIS, LK and WJM wrote the manuscript. All authors read and approved the final version of the manuscript.

## Supplementary Material

Additional file 1 **Positions of ARM in the *w*** **Mel and *w*** **Ri genomes.** Circular schemes of the *w*Ri (*Wolbachia* symbiont of *Drosophila simulans*; NC_012416; [22]) and *w*Mel genomes (*Wolbachia*, endosymbiont of *D. melanogaster*; NC_002978; [8]), showing that ARM (indicated by black bars) is equally dispersed throughout the genomes. Click here for file

Additional file 2 **Detailed information on *Drosophila *** **and *Glossina *** **specimens used in this study.** First column refers to the abbreviated code used for each specimen in text, figures and figure legends. Last column lists reference and/or collector’s name [31,11-34,12]. Click here for file
