# Supplementary figures and images for: More than fishing in the dark: PCR of a dispersed sequence produces simple but ultrasensitive Wolbachia detection
Source: BMC Microbiol. 2014 May 12;14:121. doi: 10.1186/1471-2180-14-121 (PMC4029913; doi:10.1186/1471-2180-14-121)

## Slide 1
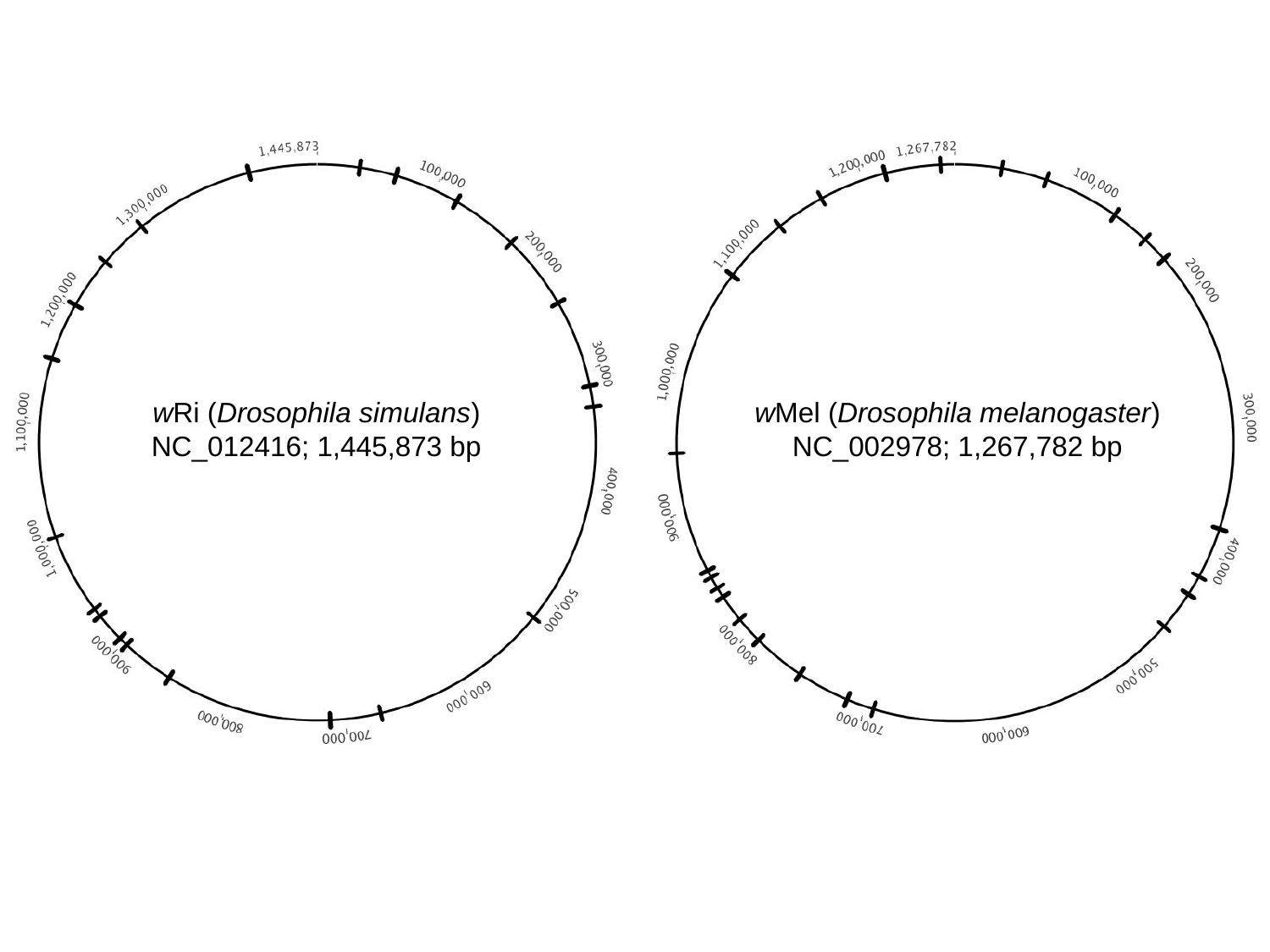

wRi (Drosophila simulans)
NC_012416; 1,445,873 bp
wMel (Drosophila melanogaster)
NC_002978; 1,267,782 bp

Supplement: Additional file 1 — Positions of ARM in the w Mel and w Ri genomes. Circular schemes of the wRi (Wolbachia symbiont of Drosophila simulans; NC_012416; [22]) and wMel genomes (Wolbachia, endosymbiont of D. melanogaster; NC_002978; [8]), showing that ARM (indicated by black bars) is equally dispersed throughout the genomes. [file 1471-2180-14-121-S1.pptx]
