# Supplementary material for: More than fishing in the dark: PCR of a dispersed sequence produces simple but ultrasensitive Wolbachia detection
Source: BMC Microbiol. 2014 May 12;14:121. doi: 10.1186/1471-2180-14-121 (PMC4029913; doi:10.1186/1471-2180-14-121)
Supplement: Additional file 2 — Detailed information on Drosophila and Glossina specimens used in this study. First column refers to the abbreviated code used for each specimen in text, figures and figure legends. Last column lists reference and/or collector’s name [31,11-34,12]. [file 1471-2180-14-121-S2.docx]

| Code in figure | Full name of strain | Species | Clade | Group | Reference/collector |
| --- | --- | --- | --- | --- | --- |
| AM1/AM2 | Amazonian semispecies, AM | *Drosophila paulistorum* | New world | *willistoni* group | [31] |
| CA1/CA2 | Centroamerican semispecies, CA | *Drosophila paulistorum* | New world | *willistoni* group | [31] |
| OR | Orinocan semispecies, OR | *Drosophila paulistorum* | New world | *willistoni* group | [31] |
| A/O | F1 hybrid from AM ♀ crossed to OR ♂ | *Drosophila paulistorum* | New world | *willistoni* group | [11] |
| *Dw*^+^ | Jaton Sacha, JS6.3 | *Drosophila willistoni* | New world | *willistoni* group | [32]; P. O'Grady |
| *Dw*^-^ | Ipitanga, WIP4 | *Drosophila willistoni* | New world | *willistoni* group | [32]; W. Heed |
| *Dm*^+^ | Harwich, H2 | *Drosophila melanogaster* | Old world | *melanogaster* group | [33] |
| *Ds*^+^ | Tucson, Arizona, Sim/Tuc1 | *Drosophila simulans* | Old world | *melanogaster* group | M. G. Kidwell |
| *Ds*^-^ | Nouméa TC | *Drosophila simulans* | Old world | *melanogaster* group | [34] |
| *Gmm* | *Glossina morsitans morsitans* | *Glossina morsitans morsitans* | - | *morsitans group* | Insect Pest Control, FAO/IAEA Vienna |
| *Gsw* | *Glossina swynnertoni* | *Glossina swynnertoni* | - | *morsitans group* | Insect Pest Control, FAO/IAEA Vienna |
| *Gs*/*Gm* | F1 from hybrid *Gsw* ♀ crossed to *Gmm* ♂ | *Hybrid* | - | *morsitans group* | [12] |
